# Comparing Effectiveness of Active and Passive Client Follow-Up Approaches in Sustaining the Continued Use of Long Acting Reversible Contraceptives (LARC) in Rural Punjab: A Multicentre, Non-Inferiority Trial

**DOI:** 10.1371/journal.pone.0160683

**Published:** 2016-09-01

**Authors:** Waqas Hameed, Syed Khurram Azmat, Moazzam Ali, Muhammad Ishaque, Ghazunfer Abbas, Erik Munroe, Rebecca Harrison, Wajahat Hussain Shamsi, Ghulam Mustafa, Omar Farooq Khan, Safdar Ali, Aftab Ahmed

**Affiliations:** 1 Marie Stopes Society, Research, Monitoring and Evaluation Department, Technical Services, Karachi, Sindh, Pakistan; 2 Department of Uro-Gynecology, University of Ghent, 9000 East Flanders, Belgium; 3 Department of Paediatric Emergency Medicine, The Hospital for Sick Children, Toronto, Ontario, Canada; 4 Department of Reproductive Health and Research, World Health Organization, Geneva, Switzerland; 5 Marie Stopes International, Research, Monitoring and Evaluation Department, London, United Kingdom; University of Pittsburgh, UNITED STATES

## Abstract

**Background:**

The use of long-acting reversible contraceptive (LARC) methods is very low in Pakistan with high discontinuation rates mainly attributed to method-related side effects. Mixed evidence is available on the effectiveness of different client follow-up approaches used to ensure method continuation. We compared the effectiveness of active and passive follow-up approaches in sustaining the use of LARC—and within ‘active’ follow-up, we further compared a telephone versus home-based approach in rural Punjab, Pakistan.

**Methods:**

This was a 12-month multicentre non-inferiority trial conducted in twenty-two (16 rural- and 6 urban-based) franchised reproductive healthcare facilities in district Chakwal of Punjab province, between November 2013 and December 2014. The study comprised of three groups of LARC clients: a) home-based follow-up, b) telephone-based follow-up, and c) passive or needs-based follow-up. Participants in the first two study groups received counselling on scheduled follow-up from the field workers at 1, 3, 6, 9, and 12 month post-insertion whereas participants in the third group were asked to contact the health facility if in need of medical assistance relating to LARC method use. Study participants were recruited with equal allocation to each study group, but participants were not randomized. The analyses are based on 1,246 LARC (intra-uterine contraceptive device and implant) users that completed approximately 12-months of follow-up. The non-inferiority margin was kept at five percentage points for the comparison of active and passive follow-up and six percentage points for telephone and home-based approach. The primary outcome was cumulative probability of method continuation at 12-month among LARC users.

**Results:**

Women recruited in home-based, telephone-based, and passive groups were 400, 419 and 427, respectively. The cumulative probability of LARC continuation at 12 month was 87.6% (95% CI 83.8 to 90.6) among women who received home-based follow-up; 89.1% (95% CI 85.7, 91.8) who received telephone-based follow-up; and 83.8% (95% CI 79.8 to 87.1) who were in the passive or needs-based follow-up group. The probability of continuation among women who were actively followed-up by field health educators—either through home-based visit or telephone-based follow-up was, 88.3% (95% CI 85.9 to 90.0). An adjusted risk difference of -4.1 (95% CI -7.8 to -0.28; p-value = 0.035) was estimated between active and passive follow-up. Whereas, within the active client follow-up, the telephone-based follow-up was found to be as effective as the home-based follow-up with an adjusted risk difference of 1.8 (95% CI -2.7 to 6.4; p-value = 0.431).

**Conclusion:**

A passive follow-up approach was 5% inferior to an active follow-up approach; whereas telephone-based follow-up was as effective as the home-based visits in sustaining the use of LARC, and was far more resource efficient. Therefore, active follow-up could improve method continuation especially in the critical post-insertion period.

## Introduction

Galvanised by the Millennium Development Goals (MDGs), many countries have achieved success in meeting demand for family planning (FP)[1,2]. Nonetheless, while making efforts to expand access for family planning, it is equally important to take into account the factors that reduce contraceptive prevalence rates such as contraceptive side-effects, method failure, discontinuation, and switching which may have negative consequences on health outcomes[3,4]. These issues are of critical importance for couples and for the programmes and policies that aim to improve sexual and reproductive health[3].

The 2015 demographic health survey (DHS) analyses of lower-income countries show that, within the first year of use, 9 percent of women discontinue using contraceptive implants, 15 percent discontinue IUDs, and 32 percent discontinue injectables. While these rates are lower than the 40 percent of women who discontinue non-LARC modern methods in the first year[5], but these rates are still unacceptably high. The most common reason for discontinuation (for all contraceptives) is reported to be method related side-effects and health concerns followed by inconvenience, and desire for a more effective method[3,5].

Generally, in community-based interventions, the home-based visits by community health workers (CHWs) are linked to improved maternal and new born health outcomes[6]. Similarly, in the context of family planning, good counselling and follow-up services may improve adoption of contraceptives, continuation and switching behaviours[7–11]. However, research studies have shown mixed results regarding the different follow-up approaches (scheduled, unscheduled, clinic based or telephone-based) used to ensure continuation rates of short- and long-acting family planning methods in different settings[12–15]. For example, higher frequency of scheduled follow-up visits (6 weeks, 3, 6, and 12 months after IUCD insertion) was found to not be effective in preventing discontinuation, pregnancies and expulsion. In another setting, telephonic interaction between staff and teenage FP users showed no effect on regularity of contraceptive use and pregnancy rates.

Pakistan, which is currently the sixth most populated country in the world, has had limited achievements in managing population growth–contraceptive use in particular has remained stagnant[16–18]. The most commonly used methods are condoms (8.8%), followed by female sterilisation (8.7%) and withdrawal (8.5%) whereas the use of long-acting reversible contraceptive is negligible (2.3%) and has remained unchanged between the 2006–07 and 2012–13 DHS surveys[16]. According to the latest DHS, 37% of women in Pakistan discontinue the use of any method within 12 months whereas IUD related discontinuation rate stands at 25%. The most prominent reason for discontinuation of IUD is side-effects (64%)[16]. Two other key obstacles that deter women to use all contraceptive methods, are husband’s opposition and social or cultural unacceptability[19].

## Rationale

Marie Stopes Society (MSS), an affiliate of Marie Stopes International in Pakistan, implemented a research study which aimed at promoting the use of modern contraception [especially long-acting reversible contraceptive (LARC)] by social franchising of private health providers and providing vouchers to the communities that these health facilities serve[20]. As part of this intervention, a community health worker [designated as Field Health Educator (FHE)] is responsible to raise awareness of family planning in the community and provide follow-up for clients for side-effect management. A single FHE would cover a population of around 20,000–25,000 spread over a 3–6 kilometre radius[20]. The role of the FHE is similar to that of both Lady Health Workers (LHW) and Community Midwives (CMW). LHWs provide needs based home visits for a population of 1,000 people, and CMWs provide needs based home visits for maternal, new born and child health for a population of between 5–6,000 people. Both the LHW and CMW programmes are run by the Government of Pakistan[21,22]. The role of FHE is confined to family planning as opposed to LHW and CMWs, the catchment population of FHE is still notably higher than that of LHWs and CMWs, which can make it challenging for her to pay routine household visits to all potential clients[23]. We therefore hoped to first test the assumption that a passive follow-up approach, which is much less resource intensive, is not inferior to active follow-up for method continuation. The saved resources could otherwise be used to focus on finding women with unmet need or to attend to other contraceptive needs of women. Moreover, given the increasing number of mobile phones in the country (120 million users[24] and 87% household possess a mobile phone[16]), which is pervasive across all sections of society, we considered testing the effectiveness of using this technology, compared to the resource intensive home-based follow-up visits.

## Research Questions

We conducted this study to address two research questions: firstly, whether a passive or needs-based follow-up is as effective as an active (active defined as home-based or telephone-based) follow-up in sustaining method continuation among LARC users. Secondly, whether or not telephone-based follow-up is as effective as physical home-based follow-up (gold standard) in sustaining method continuation among LARC users.

## Materials and Methods

### Study design and settings

This 12-month multicentre non-inferiority trial was embedded within a larger community-based experimental study[25]. The larger study aimed to evaluate the effectiveness of demand-side financing approaches in promoting the use of modern contraceptive methods[20]. We conducted this non-inferiority trial at 20 selected franchised reproductive healthcare facilities from a total of 22. Two facilities were excluded due to very low LARC client flow. Of the 20 selected healthcare facilities, 15 (led by mid-level provider) were based in rural areas and 5 (led by medical doctor) were based in peri-urban areas in Chakwal district of Punjab province in Pakistan. This study was implemented between November 2013 and December 2014 including a five month recruitment period. The final follow-up interview was conducted in December 2014, as this was when the larger study concluded. Hence, participants had a varied follow up period ranging from approximately 9 months to a maximum of 14 months–while mean duration of participants being in the study was 12 months (see Table 1).

**Table 1:** Number and percentage distribution of participants by study duration.

Duration in the study (months)	Number of participants	Percentage
**>9–10**	97	7.8
**>10–12**	622	49.9
**>12–13**	451	36.2
**>13–14**	76	6.1
**Total**	**1246**	**100.0**

### Follow-up approaches and intervention

The study comprised of three groups based on the stated approaches used for clients follow-up: a) home-based follow-up; b) telephone-based follow-up; and c) passive or needs-based follow-up. The clients recruited under category ‘a’ received a scheduled follow-up visit at their household by an FHE at 1, 3, 6, 9, and 12 months. Similarly, clients recruited under category ‘b’ received a telephone call from their FHE at a scheduled frequency similar to category ‘a’. Moreover, clients in both of these categories could also seek medical advice from their FHE or service providers based on need (unscheduled contact, either by phone or more usually in-person as service providers do not usually share contact details with clients). The clients recruited under ‘passive or needs-based follow-up’ did not receive any scheduled intervention from an FHE but could seek assistance from an FHE or service provider in the case of a medical need such as a suspected pregnancy, medical problem, method complication, or desire to remove their IUCD and/or implant. Categories ‘a’ and ‘b’ were considered as the active group whereas category ‘c’ was considered as the passive group. During scheduled and unscheduled contacts FHEs provided counselling to participants on side-effect management based on an adapted manual used in the Family Advancement for Life and Health (FALAH) project[26]. FHEs were reimbursed for household visits and telephone call expenses.

### Sampling strategy

In order to test non-inferiority between passive and active (household + telephone) groups, a sample of 1,262 was estimated by keeping the non-inferiority margin of 5 percentage points difference in cumulative probability of LARC use at 12 month, with 80% power, and 0.05 level of significance. The sample was equally distributed across the three study groups. As we had to omit the needs-based group for testing non-inferiority between telephone and home-based approaches, the non-inferiority margin was estimated slightly higher at 6 percentage points while holding other parameters constant. The sample calculation was performed using PASS version 11.0.

During the conceptualisation phase, preliminary data collected at the study sites through FHEs revealed that 40% of LARC users had access to either their own or their husbands’ mobile phone. Hence, participants were not randomised to study groups for a number of reasons—stemming from the low proportion of LARC users with access to phones. Since this trial was nested within a larger study, and thus had to conform to the larger trials budget and time frames, it was not possible to double the time frame and only recruit phone owners to allow for random allocation of study participants^32^. We enrolled a total of 1,261 women aged 15–49 years, employing a consecutive sampling technique, who received long-acting reversible contraceptive (intra-uterine device or implant) methods from the study sites and residing in the demarcated catchment area. It is pertinent to note that as per national health policy, implant insertion and/or removal services were only provided at urban-based health facilities that were led by a medical doctor where IUCD services were available at all selected health facilities. The LARC sample was equally distributed across all 22 study sites and it took approximately five months to recruit the desired sample. Prior to enrolment, participants were provided with study details and asked to voluntarily choose one of the three groups and to sign a written informed consent form. Clients who didn’t have access to mobile phones were given options to either join the passive group or the home based follow up group. Moreover, upon the achievement of the desired sample in any specific group–the group was eliminated as an option for subsequent clients. To keep a track of enrolment numbers service providers were given standard templates, where basic demographic and follow up data only was collected. While receiving their contraceptive method, all the study participants were provided standard counselling by the service providers regarding contraceptive method, including their effectiveness, possible side effects, risks and benefits, and what to do or where to go in case of experiencing any side effect^32^. Table 2 presents participants’ enrolment and response rate at the end of study.

**Table 2:** Participant’s enrolment and response rate at 12 month.

	Participants’ enrolment		
	Active follow-up group		Passive or needs-based follow-up group
	Home-based	Telephone-based	
**Baseline interviews**	409	424	428
**Follow-up interviews at 12 month**	400	419	427
**Participants’ response rate at end of study: %**	97.8%	98.8%	99.8
**Cumulative continuation at 12 month**^a^	351/400 (87.8%)	369/419 (88.1%)	358/427 (83.8%)

^a^ unweighted cumulative continuation probability

### Data Collection

At baseline (Day 0 interview at the time of recruitment), we collected information on participant’s demographic and reproductive history, fertility intentions, source of information about service providers, reasons for choosing LARC and information provided by the service provider regarding method received. At the 12-month follow-up interview, participants were asked if they were still using the same contraceptive method, or if they had stopped using it. All study participants were asked about side effect experiences. In addition, participants who reported method discontinuation were asked about the reasons for discontinuation, place of IUCD/implant removal, whether they switched to another contraceptive method. Finally, participants were also asked about their level of satisfaction with the services, counselling sessions conducted by FHEs during household visits and telephone contact with clients. To minimize bias, the interviews at 12 months were conducted by newly hired enumerators, who were trained to administer the questionnaire by the research team, rather than the FHEs who conducted the baseline data collection. We made three attempts to contact the participant for 12-month follow-up interviews; the participant was considered lost to follow-up, if all attempts of tracing the client failed. Data was captured on paper forms during face-to-face interviews and later double-entered in EpiData version 3.1.

### Statistical Analysis

We performed statistical analyses with the use of Stata software, version 11.2 (StataCorp. 2009. Stata Statistical Software: Release 11. College Station, TX: StataCorp LP.). The level of significance (alpha) was set at 0.05. We used means, standard deviations, frequencies, and percentages to describe the demographic characteristics of study participants. One-way ANOVA and chi-square test were performed for continuous and categorical data, respectively. Women in the active and passive groups were excluded from the analysis if they were lost to follow-up at the 12-month visit for missing the primary endpoint variable.

To test the hypotheses, risk differences with 95% CIs were calculated for the primary endpoint using a binomial model (generalized binary regression technique) with an identity link. Given the non-random allocation of participants to the study groups, we estimated adjusted risk differences to account for possible differences in sample characteristics such as women’s age, number of children, women’s and husband’s education, type of contraceptive method, decision for method uptake (self or jointly with others), side effect experiences (yes/no), and duration (in months) of participation in the study (>9–10, >10–12, >12–13, >13–14). The intention-to-treat population was all LARC users with data for the primary outcome except for those who withdrew consent to participate. The per-protocol population was the intention-to-treat population excluding women who had major protocol violations (e.g. crossover in assessment method). In this study, as no violation of protocol was reported, intention-to-treat analysis was used. Robust standard errors were estimated to account for clustering effect and possible correlation among participants receiving services from same health facilities. Finally, because the sample was equally distributed across study sites, we used weights to account for varying client flow.

#### Ethical consideration

All respondents were informed about follow-up procedure and their study rights. No personal information was entered in the database that could be used to identify specific individual. The study protocol was approved by National Bioethics Committee (NBC) Pakistan. Ref: No. 4-87/12/NBC-92/RDC/3548 before recruitment was initiated^27, 32^. All survey participants provided written informed consent.

## Results

### Characteristics of the participants

Characteristics of the study participants are presented in Table 3 for each of the study groups separately. Overall, most of the characteristics were similar across all three groups except for women’s age, age of last child, and type of method use. The mean age of participants was 31 years with three living children. With regard to health behaviours, nearly half of study participants used a contraceptive method in the last 3 months prior to receiving LARC. The average travel time to the health facility was 30 minutes and approximately 98% received LARC through vouchers. No significant differences were observed in any of these health behaviours.

**Table 3:** Characteristics of study participants according to study group.

Characteristics	Home-based follow up	Telephone-based follow up	Passive or needs-based follow up	p-value
	n = 400	n = 419	n = 427	
	% (SD)	% (SD)	% (SD)	
**Age of women in years**				0.03
**Mean (SD)**	31.0 (4.8)	30.3 (4.7)	31.1 (5.1)	
**Number of living children**				0.20
**Mean (SD)**	3.3 (1.5)	3.1 (1.6)	3.2 (1.5)	
**Women’s education**				0.49
**Illiterate**	42.4	39.5	47.2	
**Literate**	57.6	60.5	52.9	
**Husband’s education**				0.38
**Illiterate**	17.5	18.4	21.5	
**Literate**	82.5	81.6	78.5	
**Age of last child in years**^a^				0.01
**Mean (SD)**	2.7 (2.7)	3.2 (2.7)	3.0 (2.8)	
**Status of contraceptive use during last 3 months prior to insertion**				0.08
**Yes**	38.0	54.9	54.9	
**No**	62.1	45.1	45.1	
**Travel time to study facility (in mins)**^b^				0.21
**Mean (SD)**	31.1 (21.5)	28.7 (17.9)	29.8 (17.4)	
**Type of method used**				<0.001
**Implant**	8.7	22.8	12.1	
**IUCD**	91.4	77.2	87.9	

Missing values

^a^10

^b^68

### Participants’ health seeking behaviours and satisfaction with services

Table 4 describes the follow up information of the study participants. Approximately one in four women reported to have experienced side effects across all three study groups. Among those, ‘bleeding’ and ‘pain’ were the most common side effects experienced by LARC users. More specifically, bleeding was comparatively higher (45.5%) in the home-based group as opposed to 37% (approximately) in other two groups. On the contrary, 21.1% of participants in the passive group reported pain, followed by 14.9% in telephonic group and 12.5% in home-based. Moreover, of those who experienced side effects over 75% sought medical assistance for the treatment. Nearly 75% of women, who chose to have their IUCD/implant removed, in the active group received removal services from study providers compared to 52% in the passive group. The results also showed some suggestion (p = 0.0853) that clients who were followed up were also more likely to switch to another method in the case of discontinuation with 23.2% of the discontinuers in the passive group switching to another method compared to 30.8% and 43.0% in the home and telephone based groups.

**Table 4:** Follow up information and level of satisfaction with the services among study participants by study groups.

Characteristics	Home-based	Telephone-based	Passive or needs-based	p-value
	n = 400	n = 419	n = 427	
	%	%	%	
**Decision making for method uptake**				0.006
**Self**	32.4	43.7	37.9	
**Jointly with others**	67.6	56.3	62.1	
**Side effect experiences**				0.704
**No**	75.9	74.1	73.2	
**Yes**	24.1	25.9	26.8	
**Type of side effect experiences**				0.780
**Pain**	12.5	14.9	21.1	
**Itching**	5.3	8.7	9.6	
**Bleeding**	45.3	36.8	37.4	
**Sensation of disturbance at the place of insertion**	8.7	8.4	9.4	
**Weight gain**	11.9	11.4	5.6	
**Infection**	7.4	8.5	8.6	
**Others**	8.9	11.3	8.3	
**Seeking medical help for treatment of side effect (amongst those who experienced side effects)**				0.672
**Yes**	78.4	80.5	75.3	
**Not received or no need for medical treatment**	21.6	19.5	24.7	
**Reason for method discontinuation**				0.067
**Pregnancy desire**	31.4	31.9	28.2	
**Expulsion**	6.9	1.8	3.2	
**Opposed by husband and/or in-laws**	12.2	2.9	26.7	
**Side effects**	31.0	45.4	26.0	
**Others**	18.4	18.0	15.9	
**Place of getting IUCD/implant removal services**				0.008
**Study franchised provider**	76.6	77.5	51.9	
**Public sector provider**	11.4	8.6	32.9	
**Other private**	5.1	12.1	12.0	
**Expulsion**	6.9	1.8	3.2	
**Method switching post IUCD/implant removal**				0.0853
**Yes**	30.8	43.0	23.2	
**No**	69.2	57.0	76.8	
**Satisfaction**				
**Would recommend service to others**	92.9	92.8	88.9	0.0576
**Would recommend provider to others**	97.7	97.6	94.5	0.0170
**Provider attitude**	99.7	98.4	98.2	0.1129
**Follow-up care**	95.4	96.8	85.4	<0.001
**Feedback about follow-up mechanism (active group only)**				
**Comfortable receiving visits/calls from FHE**	99.7	98.1	-	0.0651
**Visit/Call timing was convenient to women**	99.7	99.3	-	0.9607
**Women consulted by FHE to set convenient time for follow-up**	91.4	81.7	-	0.0003
**Time spent by FHE (phone or in-person) was adequate**	99.1	98.6	-	0.5101
**Satisfied with the information provided by FHE**	99.1	98.0	-	0.1943
**Information provided by FHE was found to be useful**	93.1	90.8	-	0.0646

Reports of satisfaction with different aspects of services were very high across all three groups. Additional questions were asked to the participants that belonged to the active group regarding follow-up mechanism. Among women groups who received either a home-based visit or a telephone call, a very high percentage of the participants expressed satisfaction receiving follow-up visits from FHE, timing of visits, time spent by FHE for counseling, and satisfaction with information received and its use. Satisfaction levels were slightly lower amongst those in the passive follow up group compared to active. 89% and 95% of women in the passive follow up group reported that they would recommend the service and provider to others respectively. This compares to 93% and 98% in the active follow up groups. The only noticeable difference in satisfaction between women in the telephone-based vs home based follow up group was that 81.7% of women in the telephone based group reported that they were consulted to set a convenient time for follow-up compared with home-based group (91.4%), and 93.1% of women in the home-based follow up group found that the information that the FHE gave them was adequate compared to 90.8% in the telephone group.

### Impact of follow-up approaches on method continuation

The cumulative probability of LARC continuation at 12 month was 87.6% (95% CI 83.8 to 90.6) among women who received home-based follow-up, 89.1% (95% CI 85.7, 91.8) who received telephone follow-up, and 83.8% (95% CI 79.8 to 87.1) who were in the passive or needs-based follow-up group. The probability of continuation among women who were actively followed-up by field health educators—either through home-based visit or telephone call was 88.3% % (95% CI 85.9 to 90.0). The mean length of LARC use before removal was 8.0, 6.5 and 7.1 months in home-based, telephone-based, and passive groups respectively. An adjusted risk difference of -4.1 (95% CI -7.8 to -0.28; p-value = 0.035) was estimated between active and passive follow-up which clearly shows significant inferiority of passive follow-up relative to active follow-up in sustaining the use of LARC. In addition, within active client follow-up approaches, the telephone-based follow-up was found to be as effective as home-based follow-up (adjusted risk difference 1.8 (95% CI -2.7 to 6.4; p-value = 0.431) (Table 5). On an average, via telephone, it took 1 (SD 0.2) attempts to contact the client for follow-up counselling. The average talk time was 3.7 minutes (SD ± 2.4) with a maximum talk time of 20 minutes (results not shown)

**Table 5:** Crude and adjusted risk differences of LARC continuation at 12 months between active and passive follow-up and home-based versus telephone-based approaches.

Client follow-up approaches	Cumulative probability of continuation at 12-months	Crude risk difference	Adjusted^1^risk difference
	(95% CI)	(95% CI)	(95% CI)
**Active versus passive comparison**			
**Passive or needs-based follow-up**	83.8 (79.8–87.1)	-4.6 (-9.2, -0.01)*	-4.1 (-7.8, -0.28)*
**Active group (home-based + telephone-based) follow-up (ref)**	88.3 (85.9–90.0)	-	-
**Telephone-based versus home-based**			
**Telephone-based follow-up**	89.1 (85.7–91.8)	1.5 (-3.4, 6.4)	1.8 (-2.7, 6.4)
**Home-based follow-up (ref)**	87.6 (83.8–90.6)	-	-

* Superiority p-value: <0.05

^1^ Adjusted for women’s age, number of children, women’s and husband education, type of contraceptive method, decision for method uptake, side effect experiences, and duration (in months) of participation in the study

## Discussion

In this non-inferiority study, the data showed that the passive follow-up approach is significantly inferior, by 5.0%, to active follow-up in sustaining the use of LARC. Moreover, within the two active client follow-up approaches—our findings indicated that telephone-based follow-up works equally well to encourage LARC continuation as does home-based (gold standard) follow-up. The findings are not consistent with the earlier research conducted specifically to improve IUD continuation in the Netherlands[13]; however, it is pertinent to note that the setting, procedure and frequency of follow up was different in the two studies. The overall discontinuation rate in this study at 12 months was considerably lower than the national average[16] which may be attributed to the overall higher quality of care at the franchised clinics where services were provided under controlled research settings and rigorous monitoring. Also, the national data is a reflection of the behaviour of both public and private-sector users. The findings on discontinuation rates and its reasons were similar to the other studies conducted in Pakistan[27–29] and in other settings[7,30].

These findings are not only important for the on-going social franchising program but also at national level where the use of LARC methods of contraception is negligible, and these methods have higher discontinuation rates with the most predominant identified reason being side effects[16]. While it is important to give complete information to the family planning clients at the time of service provision, our study highlighted that active post-insertion follow-up is likely to enhance method continuation among LARC users, as well as facilitate clients to switch methods in the case of unacceptable side effects. In addition, our study presented a more resource-efficient follow-up strategy in the form of telephone-based follow-up, which was found to be equally effective as a home-based visit. In this project, FHEs were paid 57 cents (1 USD = 104.7 PKR) and 96 cents (1 USD = 104.7 PKR) for telephone-based and home-based follow-up, respectively—which clearly indicates that while telephone-based was equally effective, the cost is approximately 40% lower than a home-based visit. Hence, telephone-based follow-up is expected to save time, energy and cost in contrast to a home-based approach. Capitalising on the rapid increase in availability and use of mobile phones in the country[24], telephone-based follow-up will certainly be a convenient way to enhance method continuity for community health workers, be it FHE, Lady Health Worker, or any outreach worker who has a mandate to provide follow-up to FP clients for side effect management. This approach has the potential to reduce the amount of time and resources required for household visits—especially in rural settings. Furthermore, telephone based follow up is expected to provide CHWs as well as the users with a tool to cope with socio-cultural and geographical barriers[23,31,32] that restrict their ability to provide or access services. Lastly, this approach may help CHWs to protect the confidentiality of contraceptive users which could be compromised when clients are visited in their homes—especially in cases where women conceal their contraceptive use from other family members[33]. Above all, the unprecedented increase in the number of users of cell phone and internet technology, as well as a decline in the price of devices and services in developing countries[34–36] will increasingly facilitate programs to operationalize and sustain this approach in resource limited settings. On the whole, these approaches could prove most beneficial for improving method continuation and to ensure clients switch method in the case of intolerable side effects, thus contributing to reducing unmet need for family planning and unintended pregnancies. We encourage further research on cost-effectiveness of telephone-based follow-up approach as opposed to home-based follow-up. Moreover, the frequency of follow-up visits in this research was quite high, we propose further studies to determine the ideal number and most appropriate timing of visit post method use keeping in view of time when users are likely to experience side effect or discontinue the use of method.

Furthermore, our study provides information on other contraceptive behaviours. For example, fairly similar proportions of women sought medical assistance for side effect management across the three groups; interestingly, women in the active group were more likely to return to the same provider (who did the insertion) but they also had higher continuation rates and rates of switching to an alternative method in the case of side effects. In order to strengthen this aspect of continuation, we recommend further research to understand this phenomenon empirically. In addition, the findings showed higher method switching among clients in an active follow-up group compared with those who were in the passive group. It further substantiates that the importance of active follow-up–not only being effective in lowering method discontinuation but promoting method switching which may prevent unintended pregnancy. Method discontinuation as a result of husband and/or in-laws opposition was relatively higher in the passive group than the active follow-up groups, this may be associated with the recruitment process whereby participants were provided with options to choose the group they would like be in–and, it is very likely that women who had concealed method use from their family may have opted for passive group to protect confidentiality which may be at higher risk when being proactively followed-up FHE.

Satisfaction reported was overwhelmingly high and should be interpreted with caution. In our case, it may have been exacerbated due to strong relationship between FHE and clients that might have fostered over time. Even though this issue was minimised by having external enumerators, we suggest more robust measure methods to be used in future. Moreover, because the data were only collected at 12 month post insertion, chances of recall bias could not be ruled out. There are some limitations to this study–in particular the recruitment of participants to the three arms of the study was not randomised. This seems to have resulted in a high proportion of women in the telephone group with implants, the reason being that implants were only provided by the medical doctors who were based in the urban areas where communities were affluent and had greater access to mobile phones. This may have affected the crude results positively in favour of telephone-based group as implants have lower 12 month discontinuation rates compared with intra-uterine device[28,37]. This was adjusted for in the multivariable analyses; however, due to non-random allocation of study participants—many other observed and unobserved characteristics may vary across the study groups.

### Conclusion

Based on the findings, we conclude that the passive follow-up approach was found to be significantly inferior to active follow-up in sustaining the continued use of LARC. In addition, within active follow-up approaches, telephone-based follow-up was found to work equally well for LARC continuation as home-based (gold standard) follow-up. We propose that in order to enhance method continuity, LARC users should be actively followed-up especially in the critical post-insertion period. Also, telephone-based reminders could be used to both save resources and allow women to be followed up in a manner that maintains their privacy regarding family planning.
